# Histomorphometric method for age-at-death estimation using the anterior side of the femoral midshaft^☆^

**DOI:** 10.1016/j.jasrep.2025.105012

**Published:** 2025-04

**Authors:** Panagiota Bantavanou, Frank Siegmund, Konstantinos Moraitis, Pavlos Pavlidis, Andreas Bertsatos, Efstratios Valakos, Maria-Valeria Karakasi, Maria-Eleni Manthou, Christina Papageorgopoulou

**Affiliations:** a Laboratory of Physical Anthropology Department of History and Ethnology Democritus University of Thrace University Campus T.C. 69100 Komotini Greece; b Abt. Urund Frühgeschichtliche Archäologie, Department of History, University of Münster, Schlossplatz 2, 48149 Münster, NRW, Germany; c Department of Forensic Medicine and Toxicology, National and Kapodistrian University of Athens, Mikras Asias 75, T.C. 11527, Athens, Greece; d Laboratory of Forensic Sciences, Department of Medicine, Democritus University of Thrace, University Campus, T.C. 68100, Alexandroupoli, Greece; e Science and Technology in Archaeological and Cultural Research Center, The Cyprus Institute, Konstantinou Kavali Street 20, Agalantzia, T.C. 2121, Nicosia, Cyprus; f Department of Biology, National and Kapodistrian University of Athens, University Campus, Ilisia, T.C. 15772, Athens, Greece; g Laboratory of Histology-Embryology, Department of Medicine, Aristotle University of Thessaloniki, University Campus, T.C. 54124, Thessaloniki, Greece

**Keywords:** Microscopic analysis, Bone histology, Haversian system density, Bone remodeling, Taphonomy, Bioarchaeology, Ancient Greece, Biological anthropology

## Abstract

Age-at-death estimation on poorly preserved human skeletons is a challenging task. Histological methods in most cases can overcome this limitation and provide accurate age-at-death estimates. Yet, the standard estimation error remains at high levels. This paper presents a newly developed histomorphometric method for age-at-death estimation using femoral midshaft cross-sections from individuals of know age and sex (n = 18). For the age-at-death estimation three non-linear polynomial regression equations based on the densities of bone microstructures were generated: (a) secondary osteon population density (SD), (b) osteon fragment population density (FD), and (c) haversian system density (HD). The validation of the regression equations was performed on a test series of six modern individuals of know age-at-death and sex. The applicability of the method was further tested on individuals of modern (n = 8) and archaeological origin (n = 39) whose age-at-death was estimated through standard anthropological methods. The HD regression equation provided the highest accuracy with standard estimation error of only ±3 years. The method proved applicable on skeletal elements with different level of preservation and reliable as it successfully estimated the age-at-death of the test series. Limitations related to the asymptote phenomenon and to pathological conditions have been observed and discussed.

## 1 Introduction

Human skeletal remains are one of the most frequent findings discovered in archaeological contexts and their study is part of archaeological research. The examination of human bones offers archaeologists and anthropologists valuable information regarding the individual identities (*i.e*., age-at-death, sex) and insights on their daily life (*i.e*. diet, health). However, a variety of factors (*i.e*., burial environment, burial practice) influence bone preservation (fragmentation or missing bone elements) preventing the acquisition of such information. Thus, researchers employ analytical, genetic and histological methods in order to overcome the limitations of traditional anthropological methods in relation to those factors.

Currently, researchers utilize the morphological changes of specific skeletal elements for assessing age-at-death. However, in cases of severe fragmentation or absence of these elements, anthropological methods cannot perform efficiently, exhibiting in some cases a standard error of more than ± 10 years (Brooks and Suchey, 1990; Cunningham and Black, 2016; Franklin, 2010; Lovejoy et al., 1985a; Todd, 1920). This challenge is successfully addressed by the application of histomorphological and histomorphometric methods on undecalcified bones since their efficiency is less prone to the completeness or coherence of the skeleton. These methods are based on the study of bone microstructures (*i.e*., secondary osteons, osteon fragments), the number and morphology of which, change as the age of the individual progresses (Bell et al., 2001; Burr et al., 1990; Chen et al., 2013; Ericksen, 1991; Ortner, 1975). Histomorphometric methods have been developed on a variety of skeletal elements from the cranial (Curtis and Nawrocki, 2010), and post-cranial skeleton (Botha et al., 2019; Bouvier and Ubelaker, 1977; Cho et al., 2002; Drusini, 1987; Dudar et al., 1993; Ericksen, 1991; Goliath et al., 2016; Keough, 2007; Kerley, 1965; Pavón et al., 2010; Pfeiffer, 1992; Singh and Gunberg, 1970; Sobol et al., 2015; Stout et al., 1994; Stout and Gehlert, 1980; Stout and Paine, 1992; Thompson, 1980; Uytterschaut, 1985). A detailed summary of the published methods, including the datasets, the variables used, and their reported accuracy is presented in Table S1.

Recent studies recommend methods developed on rib cross-sections (Bonicelli et al., 2021; Cho et al., 2002; Crowder et al., 2012; García-Donas et al., 2022; Goliath et al., 2016; Karydi et al., 2022; Pavón et al., 2010; Pfeiffer et al., 2006; Stout et al., 1994) as they offer ease of sampling and (Stout et al., 1994; Stout and Paine, 1992) are subjected to less biomechanical loading than long bones (Pavón et al., 2010). This provides a more robust comparison of histomorphological measurements unscathed by inter-population variation of physical activity (Dominquez and Agnew, 2016). Histological methods of ribs utilize a diversity of microstructure measurements (*i.e*., cortical bone area, haversian canal diameters and area). Among them, osteon population density (OPD) provides the most accurate results with a standard estimation error of ±6.5 years (Pavón et al., 2010).

Ribs offer a number of benefits; however, their level of preservation is very low especially when they originate from an archaeological context. On the contrary, the femur exhibits high resistivity even in adverse conditions such as weathering and cremation, rendering the generated method applicable on poorly preserved skeletons. Additionally, the larger cortical area of the long bone provides an expanded field of view for studying the bone microstructure and their higher remodeling rate compared to ribs, renders a significant number of age-induced progressive alterations of microstructures observable (Frost, 1987; Pfeiffer et al., 2006; Pitfield et al., 2017; Robling et al., 2006; Stout and Crowder, 2012). Femoral also, sustains biomechanical loading from bipedal locomotion, which gradually changes whith age progression, leading to distinct age-related microstructural alterations (Dominquez and Agnew, 2016; Robling and Stout, 2008) that can be utilized to acquire more accurate age-at-death estimation at an individual level.

So far, two histomorphometric methods have attempted to calculate the osteon density on long bones for estimating age-at-death, although none of them included the OPD. Tompson (1979) introduced the density calculation through the “point counting” method for age-at-death estimation, resulting to an estimation error of ±7.88 years, with a standard deviation reaching up to 12 years (Drusini, 1987). Later Drusini (1987) improved Tompsons’ method decreasing the estimation error to ±3.9 years. However, a complete femur cross-section is necessary for the application of this technique compromising severely the integrity of the bone, preventing the application of other anthropological methods and restricting the application of the method only to intact bone diaphysis.

The present study aims to develop a new histomorphometric method for estimating age-at-death from long bones especially suitable for skeletal material with poor preservation and to optimize the accuracy and the estimation errors which are still quite inconsistent in other methods (Botha et al., 2019; Karydi et al., 2022; Khan et al., 2017). The method incorporates new variables such as the total density of the Haversian system (HD), the secondary osteon density (SD) and the osteon fragment density (FD) and differs from methods that use the OPD calculation on the ribs as these methods utilize the cortical area, which, due to its significant variation in long bones between sexes and ages, result in lower accuracy. To develop the method, modern and archaeological skeletal material were used and were minimally invaded, in order to ensure applicability on materials with different levels of skeletal preservation (completeness and coherence).

## 2 Materials and methods

### 2.1 The forensic and archaeological individuals

**Fig. 1:**
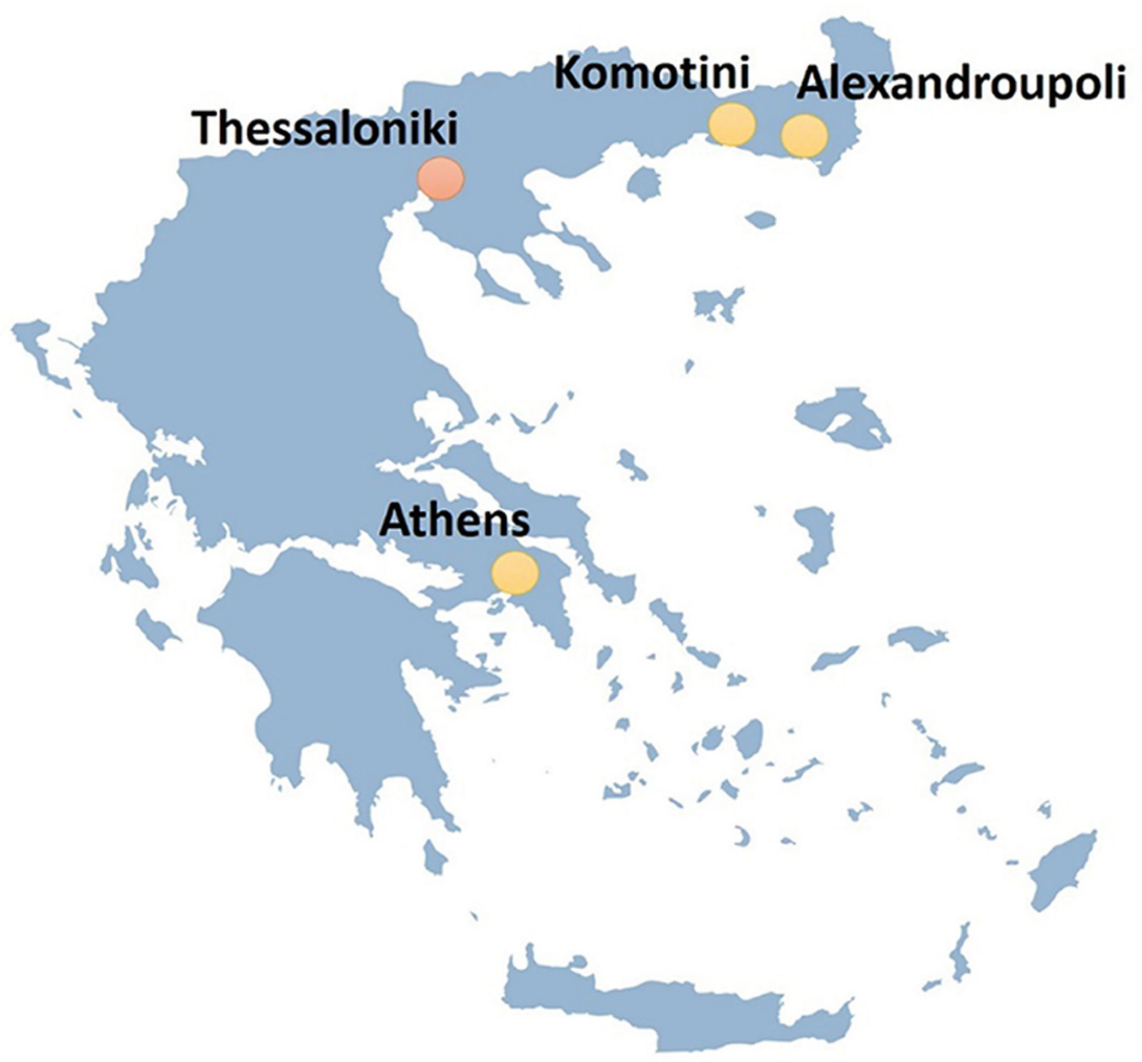
Map of Greece pointing the locations of the modern (Athens, Komotini and Alexandroupoli) and archaeological (Thessaloniki) collections of the sampled individuals.

For the generation of age-at-death regression equations, we used a reference dataset of 18 individuals from forensic cases, of known age-at-death (11 males and 7 females) with an age range of 7–86 years (Table 1). The individuals originate from the Athens Human Skeletal Reference Collection (Athens Collection), housed at the Faculty of Biology, Department of Animal and Human Physiology of the National Kapodistrian University of Athens (6 individuals) (Nikita, 2020), from the Forensic Anthropology Unit of the Medical School of Athens (16 individuals) and the Anthropological collection of Democritus University housed at the Laboratory of Physical Anthropology in Komotini (2 individuals) (Fig. 1). The model was then validated on a test series dataset, of six randomly selected individuals, also from forensic cases, of known age and sex (5 males and 1 female) with age range 47–82 years (Table 1), collected from the Forensic Anthropology Unit of the Medical School of Athens. The complete dataset was almost homogenous with the majority of the individuals being of Greek origin (15/18), and three individuals of Albanian, Pakistani and Polish origin.

The applicability of the histomorphometric method and regression equations was further tested on individuals from two contexts: a) a modern and b) archaeological. The modern series consists of eight individuals (4 males and 4 females) from the Athens Collection (2 individuals) and the Laboratory of Forensic Sciences of Democritus University of Thrace in Alexandroupoli (6 individuals) (Table 1) (Fig. 1). Sex and age-at-death were estimated for individuals from an archaeological context through established anthropological methods (described below).

The archaeological skeletal material consists of 39 inhumed individuals (20 males and 19 females) with estimated age range of 20–55 years (Table 1). The individuals originate from the ancient city of Thessaloniki in Northen Greece located on the coastline of the Thermaic Gulf (Fig. 1). The city was founded in the Early Hellenistic period (316/ 315 BCE) by Cassander brother-in-law of Alexander the Great. Cassander exploited the geographical position, the arable earth, and the commercial port transforming Thessaloniki into a significant urban and trade cross-road of the ancient Mediterranean world (Adam-Veleni, 2012; Valakopoulos, 1983). During the construction of the metropolitan subway of the city, rescue archaeological excavations uncoverd the two cemeteries of the ancient city, dated from the Hellenistic (323 BCE) to the Post Byzantine period (15th-19th c. CE) (Fig. 1). The skeletal remains included in this study were unearthed mainly from the east cemetery of the city and date to the Roman period (1st c. BC – 4th c. CE) (Acheilara, 2007; Tsimpidou-Avloniti, 2007). For detailed information see Table S2.

The study of this archaeological dataset offers significant information regarding the demographic fluctuations of an important ancient city. Thessaloniki as the capital of the Roman Imperial Province attracted merchants from all the Mediterranean world but also invaders trying to conquer and benefit from the magnitude of the city (Adam-Veleni, 2012; Soueref, 2011). Age-at-death is not only important information for reconstructing an individual’s biological identity but also for analyzing the implications of warfare and other factors on the populations’ resilience within urban contexts. The application of a histomorphometric method with high accuracy on this dataset is necessary due to the poor skeletal preservation in some parts of the ancient cemeteries. Skeletal remains dating to the Roman period usually suffer from weathering and intense fragmentation, similar to our samples. The archaeological material exhibited medium to poor state of preservation with varying degrees of integrity and completeness. Mainly the skeletons were moderately fragmented and lacked bone elements utilized in standard age-at-death estimation, e.g., pubic symphysis.

The age-at-death and biological sex of the additional modern and archaeological dataset were estimated with the standard anthropological methods. Biological sex was estimated through a combination of cranial and pelvic morphological traits (Buikstra and Ubelaker, 1994; Ferembach et al., 1980; Phenice, 1969). Age-at-death was estimated using: pubic symphysis morphology (Brooks and Suchey, 1990), and changes of the auricular surface of the pelvis (Lovejoy et al., 1985b), degree of tooth wear (Brothwell, 1981; Miles, 1962) and morphology of the sternal end of the fourth rib (Işcan et al., 1985). Macroscopical and histological analysis was conducted at the Laboratory of Physical Anthropology of Democritus University of Thrace in Greece. Approval for bone sampling and study was obtained by the Greek Ministry of Culture for the ancient individuals (395896–83422/04–6-2021) and for the modern individuals by the Democritus University Research Ethic committee. The modern individuals from the Forensic Anthropology Unit of the Medical School of Athens are retained as documented reference with appropriate ethical considerations following a forensic anthropological analysis of human remains.

### 2.2 Sampling method of the modern and archaeological material

From each individual (of modern and archaeological origin) blocks of left femur bones (or right in cases left was missing) of 1 × 1 cm were removed, from the mid-shaft anteriorly, using an electric rotary multitool with diamond surface cutting disc. The anterior part of the femur midshaft was chosen for two reasons. Firstly, because as shown by previous studies the anterior side demonstrates the most intense changes aroused by age, after the lateral side (Agnew et al., 2015). Second, because in cases of intense fragmentation, it can be better identified compared to the medio-lateral side.

### 2.3 Sample preparation for histological analysis

The protocols for histological sample preparation described below were developed in the Laboratory of Physical Anthropology, and resulted in clear and firm sections suitable for the different materials included in this study. Two different protocols were developed, one for the archaeological and one for the modern samples. The protocols were adjusted to the different degree of organic preservation and taphonomic conditions (*i.e*., fresh/dry bone, duration in the burial soil) that the samples were expose to.

The archaeological bone samples were cleansed with distilled water for 10 min in an ultrasonicator in order to repel any impurities and soil dust from the surface and micro-porosity of the bones. After cleansing, the samples were fixated in formalin (4 % NB formaldehyde, BIOGNOST CR) for 72 h in order to prevent microbial development and tissue shrinkage (Jenkins and Burg, 2003). A stepwise dehydration, in graded series of ethanol (70, 80, 90, 95, 100 % ethanol, for 48 h in each concentration) (absolute ethanol, ACS, ISO, Emsure Merck) followed in order to eliminate any residual humidity or water particles possibly trapped in the bone porosity. Such particles can destabilize the homogenization of the epoxy resin (composition resin and hardener) and prevent the proper embedding of the bone samples (Ries, 2003). Following clarification in xylene (24 h) (BioClear, BIOGNOST CR), the samples were embedded in two parts epoxy resin (resin and hardener) (EpoFix, Struers) until fully stabilized (72 h). From each embedded sample ground-sections of less than 0.5 mm were cut, with Isomet Low Speed Shaw (Buehler) with diamond surface cutting disk. The sections were thinned out to 100–50 μm with semi-automated grinder and polisher Labopol-20 (Struers) and were mounted on microscopic slides with mounting medium (BioMount DPX, BIOGNOST CR) for better visualization under the microscope light.

The majority of the modern samples were completely dry and clean so they proceeded directly to clarification in xylene and embedding in epoxy resin. In cases where fat was still preserved, the samples were defatted by infiltration in polyenzymic chemical (Endozime Premium XP, Gatex) at 60 °C (Wang et al., 2022), according to the instructions of the manufacturer. Then, they were fixated in formalin, clarified in xylene and embedded in epoxy resin as previously described. For cutting and grinding we applied the same steps as for the archaeological specimens.

### 2.4 Microscopical analysis: methodology of histomorphometry

The histomorphometric analysis was conducted on an Axioscope A1 (Zeiss) optical microscope under transmitted polarized light. Microphotographs (magnification X100) of the sections were captured with a digital camera AxioCam Icc3 (Zeiss) and imported on Fiji software (Java 1.8.0_322) (Schindelin et al., 2012) for the observation and measurements of the microstructures.

The taphonomical alterations (*i.e*., bacterial activity) observed in the thin sections of the samples were determined by the OHI (Oxford Histological Index) scale (Table S2). Samples recorded with 0, 1, and 2 of the OHI were excluded from further analysis.

The generated method was based on the Haversian system microstructures, namely the secondary osteons, the osteon fragments and their densities. Secondary osteons are termed the complete osteons (defined by an intact reversal line) that are not covered or interrupted by other osteons or primary canals. Osteon fragments are considered the remaining visual parts of older osteons that are destroyed during the remodeling process and are covered by secondary osteons or they are ruined by resorption spaces.

**Fig. 2:**
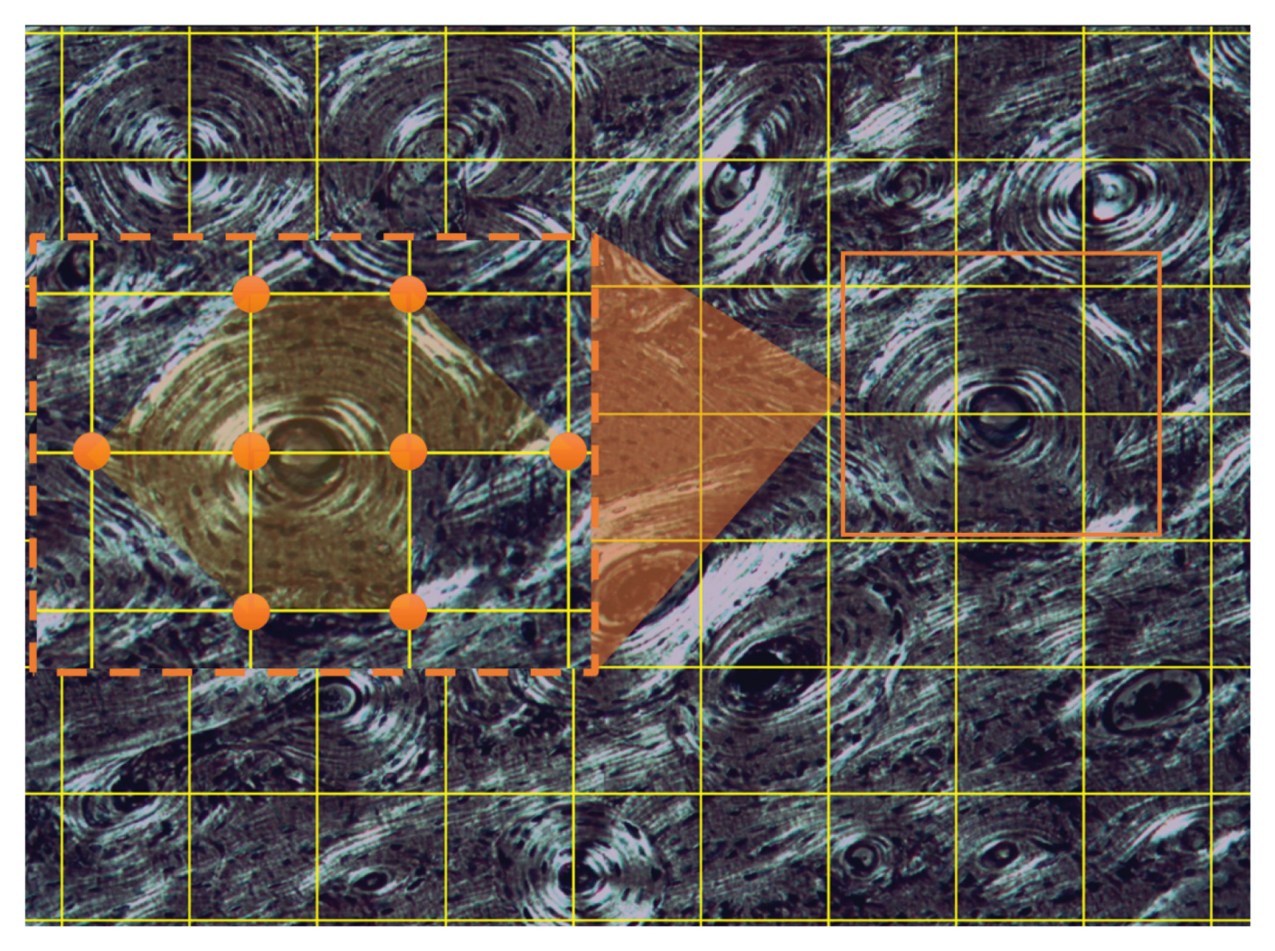
Microphotograph of the C_Athi_05 sample (Male, 44 years) from the Forensic Anthropology Unit of the Medical School of Athens, in Fiji software with a grid of squares. Magnified secondary osteon and the intersections (orange spots) for the calculation of the area of occupation (yellow filling in the squares).

From the midcortical bone (avoiding the periosteal and the trabecular parts) of each sample 10–15 random microphotographs were captured, covering each an area of 1 mm^2^. On each microphotograph the secondary osteons and osteon fragments were counted and their totals for each sample were calculated (in the sum of 10–15 microphotographs). Only osteons where 50 % or more of their total shape are observable in the image were counted. A grid was then applied, with per square area of 0.0121 μm^2^ or (0.11 μm)^2^ and the intersections of each square that surrounded and osculated the secondary osteons or the osteon fragments were marked and counted. For marking the four points of a square the osteon should cover more than 50 % of the square area. In case where less than 50 % of the square was covered the intersections were marked accordingly: 0–24 % no marked intersections, 25 %-50 % 1 to 2 marked intersections. The sum of the intersections was multiplied by the square area (0.0121 μm^2^) for estimating the area covered by the microstructures (Fig. 2). The microstructure densities were then calculated as the ratio of the total number of the microstructures to the total area they occupy. Specifically, the SD was calculated by dividing the total number of secondary osteons to the total area resulted by the sum of the intersections (per/mm^2^) for the secondary osteons. The FD was calculated by dividing the total number of osteon fragments to the total area resulted by the sum of the intersections for the osteon fragments. The HD was calculated by dividing the total number of secondary osteons and osteon fragments to the total area they all occupy as estimated by the sum of the intersections (Table 2). The calculation of the SD, FD and HD densities of femur is introduced for the first time through this study. For more details about the calculation of the densities see Example 1 in Supplementary Material.

### 2.5 Statistical analysis and regression equations

To estimate the age-at-death, single regression equations were developed, one for each microstructure density (SD, FD) and one for the total density (HD). The regression equations were generated on the reference dataset of modern individuals of known age-at-death and biological sex. Due to the small sample size the confidence intervals of the regression equations were tested with Bootstrapping. Despite the small sample size (<100), the results of the regression calculation including the confidence intervals provided solid results for future analyses at population level. Bootstrapping checks showed whether the required number of individuals is suitable for the generation of individual regression equations and whether the reference series with its appropriate variation of age groups forms a solid basis for a reliable statistical analysis. Prior to generating equations the data were examined for their normality (Shapiro-Wilk) and correlation with age-at-death (scatterplots). Their reliability was further validated on the test series of modern individuals of known age and sex. Subsequently, the method and regression was applied on both the additional modern and archaeological individuals. The age-at-death and the biological sex were estimated with standard anthropological methods. The statistical analysis of the data was conducted in R statistical software (version 2023.09.1 + 494) (R Core Team, 2023). Statistical tests (non-zero variance, Breusch-Pagan homo/heteroscedasticity, Breusch-Godfrey autocorrelation, Shapiro test for residual distribution, Bonferroni outlier test, Cook’s test for influential cases, AIC-Akaike Information Criterion for comparison of the regressions, Bootstrapping for stability) were applied for the evaluation of the regression equations. Bootstrapping was performed with the R package “boot” (Canty and Ripley, 2022).

## 3 Results

### 3.1 Analysis of forensic reference individuals

All samples (n = 18) where histologically processed and the density values were calculated. Descriptive statistics were conducted to estimate the mean, median, standard deviation and quantile values. The statistical analysis revealed that the four male individuals over 60 years old (n = 4) were affecting intensely the distribution of the osteon density values and therefore the confidence interval of the regression model (Fig. S1, S2, S3). As seen in Fig. S1-3 the elderly individuals exhibit a resemblance to the values of the middle-aged individuals, skewing intensely the data and preventing the generation of any regression model, whereas the elderly female individuals demonstrated elevated density values without disturbing the regression structure. Consequently, they were excluded from the reference sample together with two other individuals: a 77 years old female individual (C_Volhi_02) who proved to be an outlier by the Bonferroni outlier test and, a male individual (C_Athi_01) who was histologically diagnosed with osteoporosis. After the reasonable exclusion of these six individuals, we proceeded to the statistical examination and performance of regression equations on 12 individuals, equally representing males and females and without changing the age range of 7–86 years.

**Fig. 3:**
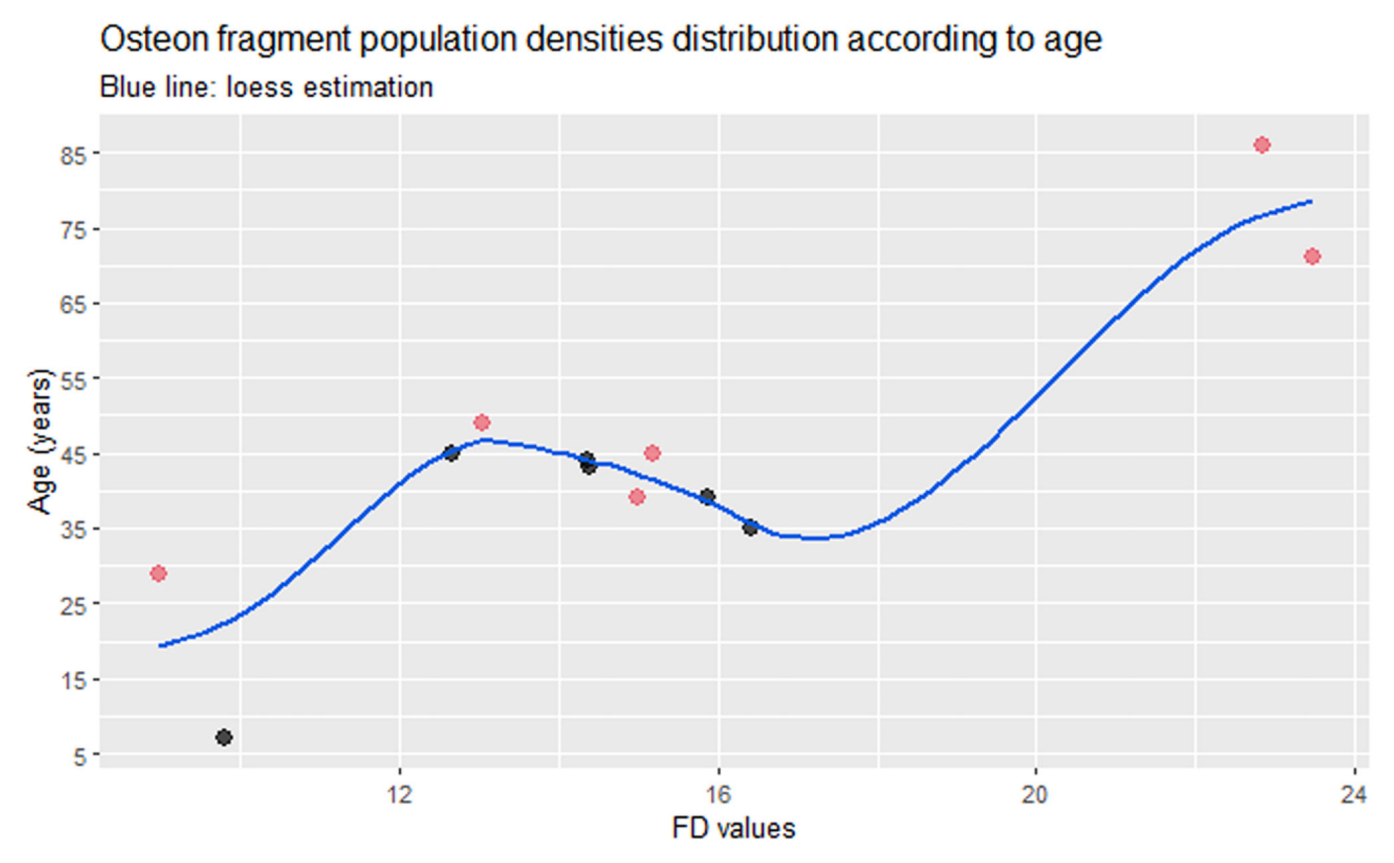
Scatterplot of FD values distribution according to the ages of 12 modern individuals of known age and sex. Males are colored with gray and females with coral.

**Fig. 4:**
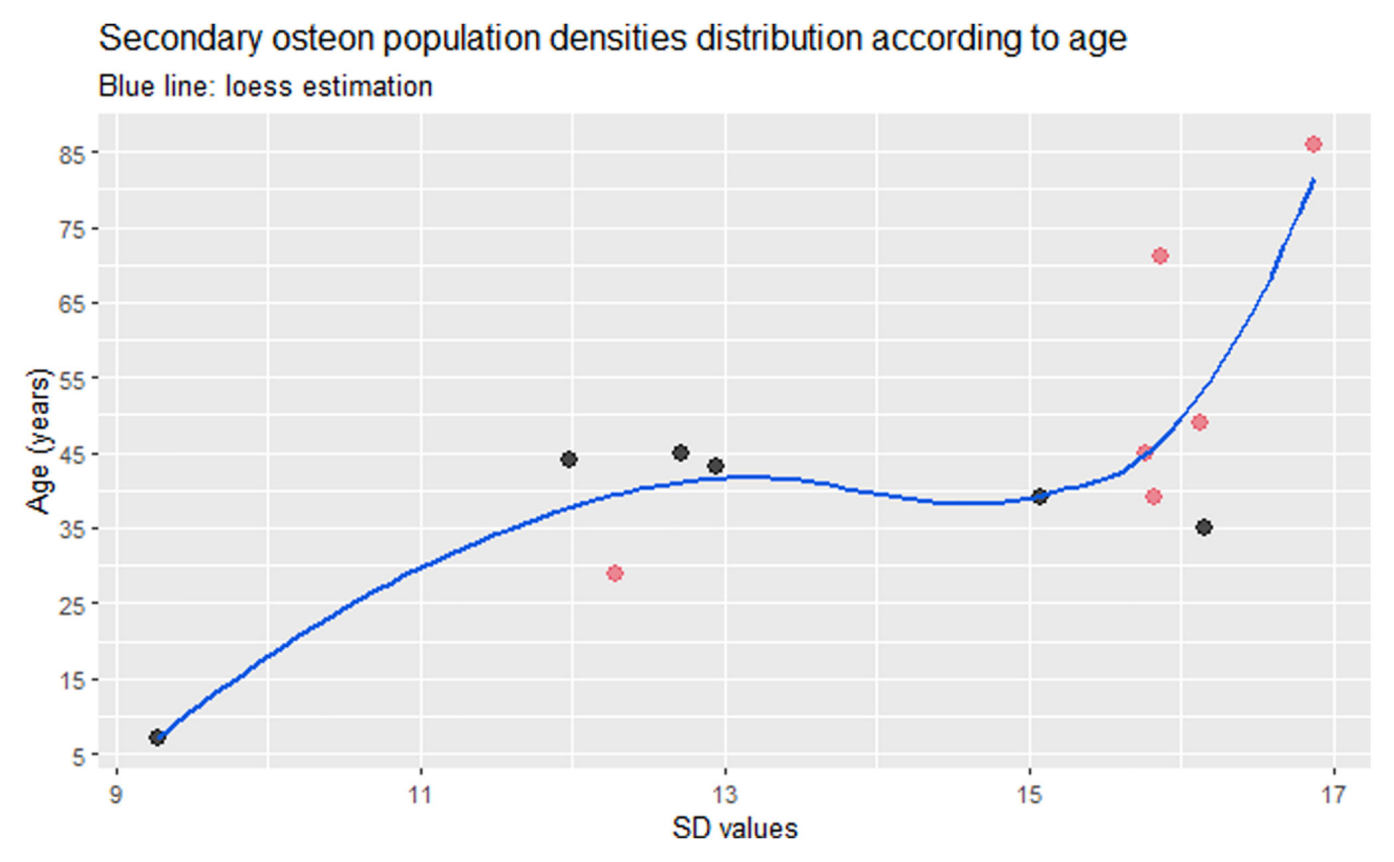
Scatterplot of SD values distribution according to the ages of 12 modern individuals of known age and sex. Males are colored with gray and females with coral.

**Fig. 5:**
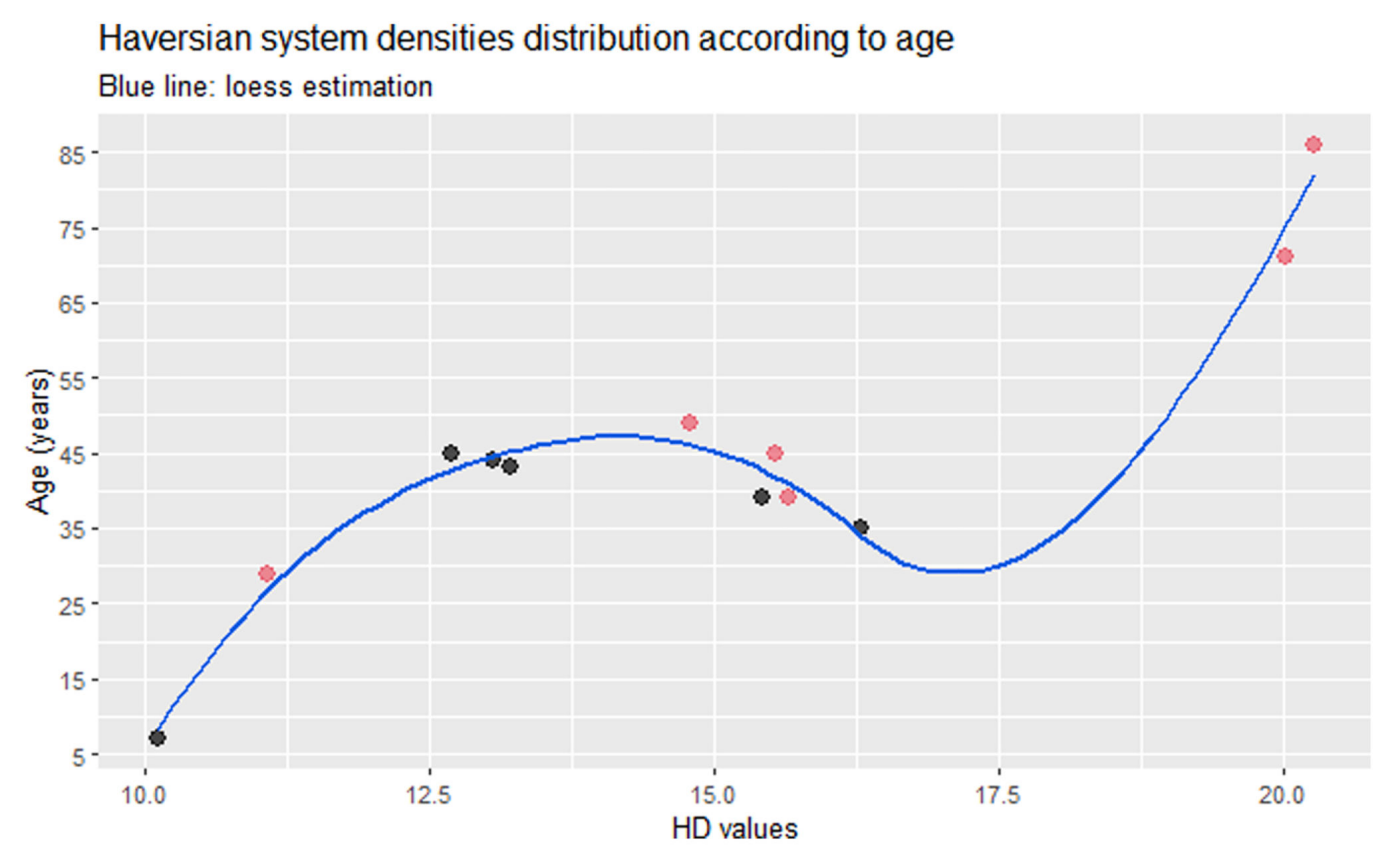
Scatterplot of HD values distribution according to the ages of 12 modern individuals of known age and sex. Males are colored with gray and females with coral.

Shapiro-Wilk normality test revealed that all density values (SD, FD and HD) were not normally distributed (p-value > 0.05). The density examination of the SD, FD and HD values showed a bimodal concentration of the individuals to the lower and higher values, less centered to the median. The distribution of the SD, FD and HD values according to age revealed a non-linear correlation, rather the observations seem to follow a curve of a 3rd degree (Figs. 3, 4, 5). Following the results, we performed single non-linear polynomial cubic regression equations.

The cubic regression equation of SD, despite the statistically significant results (coefficients p < 0.05 and regression p-value < 0.05) yielded very high (±12.67 years) residual standard estimation error (RSE). Similarly, the FD regression equation was statistically significant (p-value < 0.05) with high RSE (±10.86 years). On the contrary, the HD regression equation exhibited statistical significance (coefficients p < 0.01 and p-value < 0.01) resulting in low RSE (±3.8 years). The individual deviations ranged between 1–5 years, demonstrating the best fitting to our data (r^2^ = 0.97) (Tables 3 and 4). As SD and FD regressions did not provide the sought out results we proceeded with the evaluation only of the HD regression equation. For the performance of the HD regression equation and the estimated ages of the excluded individuals see Supplementary Table S2.

The evaluation of the HD regression equation was assessed through standard statistical assumptions. The Bonferroni test revealed no significant outliers. The Shapiro-Wilk test showed that the residuals were not violating the assumption of normal distribution (p-value = 0.28). Three individuals, in particular the two elderly females (C_Athi_08 and C_Athi_09) and the subadult individual (C_Athi_06) from the reference sample, seem to be influential as they are part of the two end points of the regression. However, Cook distances and Hat values (<1) showed that the influence was not strong enough to skew the results. Finally, non-parametric bootstrapping was performed for assessing the variance and the standard error of the HD regression equation. The HD values, from the 12 individuals of the reference dataset, were randomly sampled and replaced for 5000 replications. Bootstrapping showed stable results with R^2^ value at 0.97. The 95 % confidence interval ranges from 0.90 to 0.99, while the BCa confidence interval ranges from 0.68 to 0.99. Bootstrapping of the regression coefficients revealed that all but one (coefficient of 3rd degree slope) result in high estimation errors and high confidence intervals. These results suggest possible overfitting of the regression equation and borderline stability.

### 3.2 Results of the test series: Individuals of modern origin

The histomorphometric method was effectively applied on the test series. The HD regression equation provided similar performance with the reference sample. The age-at-death estimates of the middle-aged individuals (C_Athi_11, 16, 17) were highly accurate with a deviation of 5 years. The advanced age individuals (C_Athi_13, 14, 15) were significantly underestimated from 19-35 years (Table 5).

### 3.3 Application on additional material: Individuals of modern origin

The HD regression equation was applied was applied to 7 of the 8 modern individuals of the test series (Table 6). One individual (C_Alhi_02) was excluded due to intense bacterial activity (OHI 0) that prevented the calculation of the HD value. The regression equation performed efficiently, on the remaining individuals, and accurately estimated the age-at-death within the acceptable deviation error (1–5 years) on six out of seven. A middle-aged male individual (C_Alhi_04a) was falsely estimated for at least 25 years.

### 3.4 Population variation of HD values

Inter- and intra-age and sex variations in the HD values are observable. Middle-aged individuals exhibited a wider range of HD values starting from ~ 14 to 16 mm^2^, with no difference between sexes. Due to this fact the regression equation estimated efficiently all the middle-aged individuals. Nonadult individuals possibly exhibit a denser value range, however this study does not have sufficient data to support such a hypothesis. The old female individuals of this study exhibit higher values from the middle-aged individuals with low variability, ranging from 18 to 20 mm^2^. However, elderly male individuals diverged from the female values, exhibiting similar to the middle-aged values.

### 3.5 Application on additional material: Individuals of archaeological origin

**Fig. 6:**
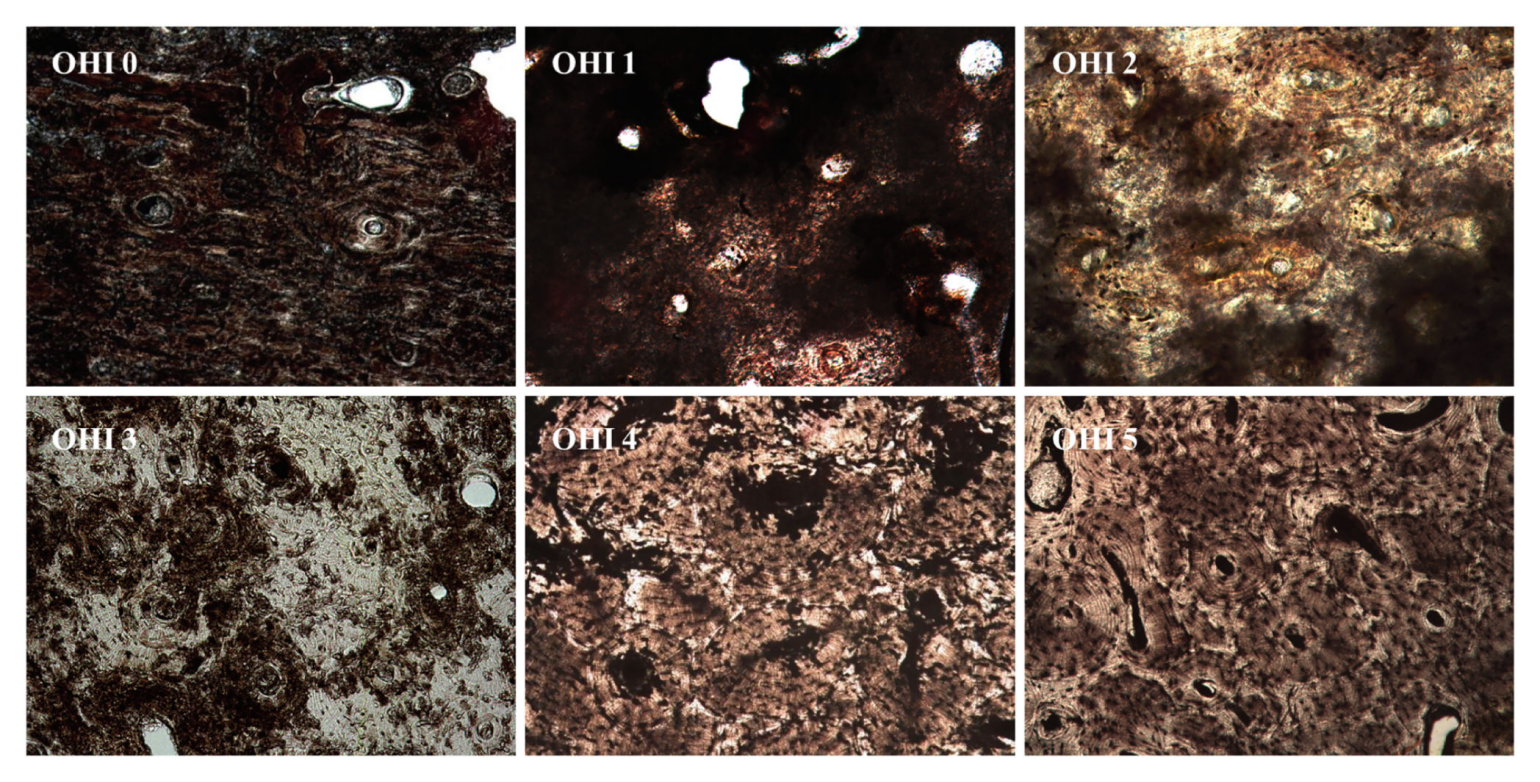
Microphotographs of individuals from ancient Thessaloniki. Each micro-image represents a scale from the OHI index based on the percentage of microstructure preservation, with 0 depicting the absolute destruction of the microstructure and 5 the complete intact microstructure.

The application of the method on the archaeological individuals proved adequate. However, bacterial activity prevented the observation of microstructures and thus the age-at-death estimation for 14 individuals. These samples were scored with 0, 1, and 2 on the OHI scale and were excluded (Fig. 6) (Table S2) reducing the sample size from 39 to 25 individuals.

The HD regression equation estimated precisely 22 out of 25 individuals of archaeological origin. Estimated ages were approximate or coincided with the anthropological age ranges with a deviation of 1–5 years with exception of three young adult female individuals (METh_08, 54, 74) whose estimated age deviated 10–20 years from the anthropological age ranges (Table 7). The Mann-Whitney-*U* test showed no sex differentiation of the estimation errors between males and females.

## 4 Discussion

The aim of this study was to generate a histomorphometric method that will accurately estimate the age-at-death of poorly preserved skeletal material from an archaeological and forensic context using femoral diaphysis sections. For this, we analysed the bone microstructure from 32 modern individuals from the Human Skeletal Reference Collection (Athens Collection), the Forensic Anthropology Unit of the Medical School of Athens, the Laboratory of Forensic Sciences of Alexandroupoli and the Anthropological collection of Democritus University and from 39 inhumed individuals from the ancient city of Thessaloniki dated in Roman era.

### 4.1 Demographic data of age-at-death of ancient Roman individuals

The archaeological sample population in this study originates from the city of Thessaloniki and dates back to the Roman period (1st c. BCE – 4th c. CE). Since the chronological estimation of the remains is significantly diverse and scattered in the time-range of Roman period, and the sample size is not representative of the entire population, demographic results should be considered with caution. According to the standard anthropological methods the age-at-death estimates of the individuals ranged between 20 to 60 years and with mean age-at-death 37.38 years old. These methods provided individual age-at-death ranges of more than 10 years span in 12 out of 25 individuals (Table 7). This large age span results from the poor skeletal preservation and specifically from the brittle character of the human remains and the destruction of significant, for age estimation, bone elements like the pubic symphysis.

The age-at-death estimates provided by the histomorphometric method corresponded with the age-ranges provided by the standard anthropological methods in the majority of the individuals (23/25). In some cases (n = 4) the age-at-death estimates deviated from the anthropological age-ranges by approximately 5–6 years, while in three individuals the age estimates deviated significantly from 10 to 20 years (Table 7). Interestingly, the results of the histomorphometric method revealed a pattern, with the age-at-death estimates tending to reach the higher ends of the macroscopical age-ranges in 11 individuals, the mean ages in seven individuals, while in four individuals the age-at-death estimates were closer to the lower age ends. Overall, the results of the histomorphometric method indicate that the individuals were slightly older than estimated by macroscopical methods, providing a mean age-at-death at 41.54 years old. This study has elucidated that histomorphometric methods are suitable and applicable in materials of different conditions of preservation, yielding highly accurate results.

### 4.2 The significance of Haversian system density (HD) in age-at-death estimation

The histomorphometric method developed in this study using femur bones, provides an excellent insight of the bone microstructure changes emerging from age. The SD, FD and HD densities are based on a different calculation method from OPD. The cortical area that is necessary for OPD is not utilized and this leads to the great advantage of minimal destruction of the bone required for the application of this methodology, in contrast with ribs and other long bone methods (*i.e*. Kerley (1965), Goliath et al. (2016), Stout and Paine (1992)) that demand the removal of a complete cross-section. On the contrary, in the Haversian system density only the Haversian area is measured, in high detail, capturing the densities fluctuation across age groups.

Regression equations of SD and FD resulted in high estimation errors and did not fit our data (Fig. S4, S5). Similar histomorphometric studies developed on ribs showed that the variables of secondary osteons and osteon fragments densities performed inadequately resulting in inaccurate estimations and underestimation of the individuals (Cho et al., 2002; Pavón et al., 2010; Stout and Paine, 1992). This is an expected outcome since the two distinct microstructures represent different remodeling phases and exhibit increased variability of the distribution (Fig. S4, S5) with no clear pattern as the individual ages (Cooke et al., 2022). In this study, the FD regression equation over- and underestimated the individuals with no clear pattern and resulted in no significance, being vulnerable to the individual variability and the asymptote phenomenon (see below). Similarly, the SD, exhibited a clear trend overestimating the younger individuals and underestimating the older individuals verifying that the secondary osteons alone are not sensitive enough to recognize the age changes.

The HD value, although it is assessed as a combination of SD and FD, provided accurate estimations (within the accepted regression error of 1–5 years), for all age groups of modern females and male individuals below 60 years old. Furthermore, the HD regression equation provided the lower RSE (±3 years) published so far from methods developed both for long bones and ribs (Cho et al., 2002; Dominguez and Mavroudas, 2019a; Dudar et al., 1993; Goliath et al., 2016; Pavón et al., 2010; Pfeiffer, 1992; Sobol et al., 2015; Stout et al., 1994; Stout and Paine, 1992; Suzuki and Maggiano, 2018). In contrast to our study, most of the OPD regression equations tend to underestimate individuals and result in higher deviations from the real ages. The HD is not influenced by the numeric variation of the microstructures, since the combination of complete osteons and osteon fragments engage all the remodeling phases and is more sensitive to age alterations (Andronowski et al., 2018).

Age, among various factors (*i.e*., remodelling rates, biomechanical loading) (Dominquez and Agnew, 2016; Suzuki and Maggiano, 2018), significantly influences osteon size (Currey, 1964; Maat et al., 2006; Martin et al., 1980; Matsuo et al., 2019; Mulhern and Van Gerven, 1997) and consequently the HD. Throughout childhood and adolescence (0–15 years) the osteons are large and irregular and the majority consists of drifting osteons that cover large areas of the cortical bone (Fig. S6A). During adulthood (20–40 years) the osteons shape gets more regular, is medium- and small-sized and characterized by larger haversian canals (Fig. S6B) (Burr et al., 1990). Finally, the osteons in elderly (>50) individuals end up being small and more circulated (Fig. S6C) (Currey, 1964; Goliath et al., 2016; Maat et al., 2006; Martin et al., 1980; Matsuo et al., 2019). Based on this, nonadult individuals have few large osteons that cover an extended bone area and their HD value is low. Whereas, as the individual gets older, the osteon size decreases, the area they occupy also decreases but their quantity is significantly increased. As a result, the density values of older individuals are high (Currey, 1964; Maat et al., 2006; Maggio and Franklin, 2019; Martin et al., 1980; Matsuo et al., 2019). Thus, the HD is more efficient for age estimation than the absolute numbers of the microstructures.

The validation on the test series of modern individuals of known age-at-death and sex proved the accuracy of the method and pointed out the weaknesses. The regression equation estimated the middle-aged individuals (C_Athi_11, 16, 17) with high accuracy, with a deviation of 3–5 years from the real age. However, the regression equation failed to estimate age in older individuals (C_Athi_13, 15), including one female (C_Athi_14). This is an expected outcome, since the method cannot estimate male individuals over 60 years old, that includes also in this case and the 58 years old male individual. The female individual of 82 years old was significantly underestimated by approximately 20 years, suggesting that age estimates in female individuals over 60 years old should be treated with caution.

The application of the HD regression equation to the additional modern individuals proved capable and reliable, as the individuals’ age-at-death were aligned with the age ranges obtained from the standard anthropological analysis. In six of the eight modern individuals age-at-death could be accurately estimated. In two male individuals (C_Alhi_02 and C_Alhi_04(1)) age could not be accurately estimated and will be further discussed below.

Despite the precision of the method, the HD regression equation demonstrated certain limitations. The statistical evaluation of the HD regression equation showed marginal stability as the minimum number of samples for single variable 3rd degree regression equations is 10, placing our sample size (n = 12) close to the lower limit. Moreover, due to the insufficient number of nonadult individuals (n = 1) in the reference sample the accurate estimation of nonadult individuals is uncertain and needs further investigation.

The HD regression equation was found inadequate to completely overcome the boundary of 60 years old, since five male individuals from the reference sample and three individuals from the test series were considerably underestimated. This result indicates that even though the application of HD has several advantages, is still susceptible, at some level, to the asymptote phenomenon. The asymptote phenomenon is reached when the cortical bone is totally covered by the Haversian system, during each remodeling phase a standardized number of secondary osteons is created destroying the underlying layers of microstructures (Andronowski et al., 2018; Dominguez and Mavroudas, 2019a; Ott, 2002; Stout and Lueck, 1995). In ribs the asymptote has been recorded to commence around age of 50 while in bones with larger cortical area, like femur, this takes place a little later (Dominguez and Mavroudas, 2019). As a result, the histological methods are less accurate in individuals of advanced age because they tend to underestimate the real age (Crowder et al., 2022). Cho and colleagues (2002) in their study of inter-population age estimation mention the low accuracy of age estimates in older individuals (>60 years) due to asymptotic OPD values.

Goliath and colleagues (2016) highlights the inadequacy of OPD as a single variable for assessing age-at-death of old individuals due to asymptote phenomenon. Our results agree with other studies as the HD values of the individuals over 60 years old were similar to that of the younger individuals (see Table S2). This is a clear observation as the majority of the older individuals (>40 years) have approximately the same average number of secondary osteons per square mm (5–7 SO#1mm^2^) (Fig. S4), although a highly diverse number of osteon fragments (Fig. S5).

The asymptote phenomenon is also highly sex-specific. Narasaki and colleagues (1990) noted that bone morphology in males was less age dependent than in females. The cortical area in males over the age of 54 years does not change whereas, in females it decreases (Ruff and Hayes, 1988). This emerges from the estrogen deficiency that females undergo after the age of 50 years, that affects, in turn, the remodeling process and leads to the thinning of the cortical and trabecular bone volume size (Seeman, 2008). The reduction in volume size results in a decrease of bone area, which is a fundamental component of the OPD. Consequently, while the cortical area continuously diminishes and the bone becomes full of small osteons that characterize the senile individuals, the OPD increases. Although, HD is not affected significantly by the volume of cortical area, it remains susceptible to the substantial degradation of cortical bone resulting from alterations in osteoclast and osteoblast activity, creating enlarged resorption spaces. This continuous change of the bone in females allows for the HD regression equation to provide accurate estimates even over the age of 60 years old. On the contrary, the male hormonic levels do not influence significantly the cortical bone structure, but lead to a progressive thinning of the trabecular bone (Seeman, 2008). As a result, the cortical area and the number of microstructures remain consistent in males over 60 years of age limiting the efficiency of HD regression equation. In our study, seven male individuals (>60 years) of the reference and test sample (including the individual who suffered from osteoporosis) were underestimated significantly, while six female individuals from the reference sample (C_Athi_08,_09 and ABHT-1,2,3,4) were accurately estimated, and two were significantly underestimated (C_Athi_14 and C_Volhi_02). However, the sample in our study is insufficiently represented by females and requires additional investigation to validate the efficacy of the findings.

Beside age and sex OPD values are affected by factors such as diet, health conditions and social status (Andronowski et al., 2018; Goliath et al., 2016; Miszkiewicz, 2016; Miszkiewicz and Mahoney, 2016; Pavón et al., 2010; Richman et al., 1979; Robling and Stout, 2008; Stout and Lueck, 1995), that are the significant pillars of healthy bone development. Therefore, pathological conditions and congenital diseases that disrupt bone homeostasis can also affect OPD values (Dominguez and Mavroudas, 2019). A male individual (C_Athi_01) of the reference sample was microscopically diagnosed with osteoporosis and therefore was excluded from the dataset. Osteoporosis is a frequent condition that appears mainly on middle and elderly adults and disturbs the remodeling procedure of the bone, creating massive resorption spaces (Barger-Lux and Recker, 2002; “Consensus Development Conference: Diagnosis, Prophylaxis, and Treatment of Osteoporosis,” 1993; Yerramshetty and Akkus, 2013). The extensive bone destruction from the osteoclasts and slow remodeling from the osteoblasts disrupts the normal structure of the cortical bone resulting in insufficient number of microstructures (secondary osteons and osteon fragments) and decreased cortical area.

Therefore, the HD values are not representative to the chronological age. Likewise, congenital and metabolic diseases can disrupt the bone’s mineralization and homeostasis (Khurana and Fitzpatrick, 2009). A young male (C_Alhi_04[1]), of the modern additional material, suffered from ankylosis spondylitis that was diagnosed during the macroscopical analysis. The bone microstructure exhibited low mineralization and abnormal growth of the Haversian system with scattered small and round osteons (Nigil Haroon et al., 2015; Zhang et al., 2003). This produced a biased HD comparable to that of a senile individual leading to false age estimation.

### 4.3 Advantages of the histomorphometric method based on HD

Overall, the generated histomorphometric method shows certain advantages that contribute to the efficiency of the method. The HD can be computed with high accuracy both from complete and small parts of cross-sections just as OPD (García-Donas et al., 2016; Iwaniec et al., 1998), offering the opportunity of minimal bone destruction. Leveraging the efficacy of this methodological approach, the degree of invasion was successfully minimized. Additionally, the possibility of additional latent error due to small bone sample is limited, as HD is not reliant to the size of the bone sample (Stout et al., 1994).

The larger cortical bone of the femur and the lower bone remodeling rate compared to the ribs allows the observation of more remodeling phases. The anterior side of the femur midshaft was carefully selected since it is identifiable even in cases of intense fragmentation, and demonstrates intense changes emerged by age (Agnew et al., 2015). Other sides of the femur have demonstrated the same (posterior side) (Maggio and Franklin, 2021) or even greater age related changes of the microstructure (lateral side) (Agnew et al., 2015). However, the sampling of a side with questionable identification (*i.e*., lateral side), constrains the application of the method exclusively to bones with better morphological preservation. Furthermore, although the posterior side has been found to demonstrate similar results with the anterior side (Maggio and Franklin, 2021) the remodeling activity of the side still generates cautions due to the biomechanical strain from the muscle attached to the linea aspera. Chan and colleagues (2006) in their research applying Tompsons’ (1979) method observed that posterior side was the only one, resulting in different age estimates. They suggest that, in addition to other factors, the mechanical loading has a substantial impact on the posterior region by regulating (increasing or decreasing) the remodelling rate according to the individuals’ physical activity (Chan et al., 2007).

Apart from securing the homogeneity of the samples, the uniformity of the histological observations was also important. This was achieved by utilizing only the Haversian system of the midcortical bone excluding the periosteum and the endosteal part close to trabecular bone. These areas have been suggested to demonstrate different structural organization (*i.e*., trabecular struts, microstructural heterogeneity) (Parfitt, 1984; Ruff and Hayes, 1988; Yeni et al., 2013), to be more susceptible to external factors (*i.e*., physical activity) (Ju and Sone, 2021; Yeni et al., 2013) to develop different growth rates, and to show significant differences in mineral density and number of microstructures (Parfitt, 1984).

Finally, a limitation that affects all histological applications is the taphonomical conditions, and specifically the bacterial activity. Intense bacterial activity was observed on the individuals of archaeological origin and on one modern individual of the additional material (C_Alhi_02). Natural decomposers significantly affected the archaeological inhumed bones (14/39), reducing the sample size from 39 to 25 individuals. Targeting the bone proteins, endogenous (gut bacteria) and exogenous (*i.e*., terrestrial, aquatic, cyanobacteria) bacteria invade in the bone structure, shaping different forms of tunneling (linear-longitudinal, budded, non-Wedl and Wedl) (Bell, 1990; Bell et al., 1996; Booth, 2017; Caruso et al., 2018; Hackett, 1981; Jans, 2014; Jans et al., 2004; Turner-Walker, 2019). Furthermore, the prolonged period of time of the skeletal remains buried in the ground and the taphonomical fragmentation of the bones, enable the access of the bacteria to the inner bone structure (Kontopoulos et al., 2019). These bacterial imprints of focal destruction do not reflect the microscopic light properly, preventing the visualization and analysis of the microstructures. Therefore, 15 individuals (14 archaeological and 1 modern) with intense tunneling were excluded from further study.

Research regarding taphonomical effects on bone microstructure is significantly limited in ancient Greek populations. Histological data from Greek sites (Kastrouly in central Greece, Archontico and Thessaloniki in northern Greece) indicate that a significant number of individuals exhibit bacterial activity. Specifically, in Thessaloniki 89 % (35/39) of the individuals exhibited focal destruction with 40 % (14/35) of those be severely damaged. Similarly, the histological preservation in Kastrouly is quite low with 60 % (9/15) of the individuals exhibiting taphonomical alteration. However, the authors do not mention the severity and extension of the destruction (Kontopoulos, 2019). Contrary to Thessaloniki and Kastrouly, only 21 % (11/52) of Archonticos’ individuals displayed focal destruction with the 63 % (7/11) of them display severe destruction (Kakasa et al., in press). Bone diagenesis, and specifically bacterial activity, is a complex procedure and multifactorial phenomenon that still poses challenges for histological research (Mavroudas et al., 2023). The histotaphonomical results from studies on ancient Greek populations indicate that sampling for histomorphometric studies should be considered with caution, as there is a high possibility that bacterial activity may affect the microstructure of the bone.

Finally, histological analysis is a feasible technique since the sample preparation is not bound to specific equipment, the consumables are not expensive and the cost can be adjusted to the laboratory financial adequacy. Using a manual method, such as Maat et al. (2001) the cost can be sufficiently low, while methodologies with sophisticated equipment cost more. However, the authors recommend the utilization of machinery for high quality histological sections.

## 5 Conclusions

The results of this study indicate that the HD value proved a reliable variable for age-at-death estimation using the anterior femur midshaft producing the lower RSE published so far both from femur and ribs. The generated HD regression equation resulted accurate estimates for the individuals of both sexes with exception of the individuals above 60 years of age for whom the human physiological mechanisms stand as an obstacle and needs further consideration. The generated histomorphometric method and the HD regression equation performed efficiently on human skeletal material of modern and archaeological origin. Further study will improve the results upon subadult and senile individuals as also intra-population differences. The histomorphometric method enabled the age estimation of individuals with poor skeletal preservation shedding light to the demography of people living in Greece during the Roman Era. Such methods can contribute to the palaeodemographic analysis of past societies.

## Supplementary Material

Supplementary data to this article can be found online at https://doi.org/10.1016/j.jasrep.2025.105012.

Supplementary Material

## Figures and Tables

**Table 1:** Biological sex and age-at-death from the reference, test and additional datasets of individuals of modern and archaeological origin. The age-at-death and biological sex of the contemporary reference and test samples are documented, while the biological sex and age-at-death of the additional datasets of modern and archaeological origin were estimated with standard anthropological methods.

Datasets	Male	Female	Total	Age range (years)
Reference dataset: documented modern individuals	11	7	18	7–86
Test dataset: documented modern individuals	5	1	6	47–82
Additional dataset: modern individuals	4	4	8	25–59
Additional dataset: archaeological individuals	20	19	39	20–55
**Total**	**40**	**31**	**71**	

**Table 2:** Estimation of the area and the density of each microstructure.

	Formula for area estimation
Area of secondary osteons =	Total number of ^a^SO intersections * 0.11^2^
Area of osteon fragments =	Total number of ^b^OF intersections * 0.11^2^
Total osteonal area =	Total number of OF + SO intersections * 0.11^2^
	**Formula for density estimation**
^c^SD =	Total number of SO / Area of secondary osteons
^d^FD =	Total number of OF / Area of osteon fragments
^e^HD =	Total number of osteons / Total osteonal area

a SO = secondary osteons.

b OF = osteon fragments.

c SD = secondary osteon density.

d FD = osteon fragment density.

e HD = Haversian system density.

**Table 3:** Summary of the three non-linear cubic regression equations, generated on the reference samples of modern individuals of known age and sex.

Variable	Regression equation	RSE	R^2^	p-value
^a^SD	y = 506.3154*x – (39.4817*x^2^) + (1.0158*x^3^) – 2103.4885	12.67	0.69	0.017*
^b^FD	y = 57.54242*x – (3.66573*x^2^) + (0.07812*x^3^) – 259.72227	10.86	0.77	0.005*
^c^HD	y = 290.65337*x – (19.48360*x^2^) + (0.42995*x^3^) – 1384.67423	3.802	0.97	0.00*

* a = 0.05.

^a^ Secondary osteon population density (SD), ^b^Osteon fragment population density (FD), ^c^Haversian system density (HD).

**Table 4:** Real ages of the modern individuals of the reference sample in comparison with the estimated ages from the HD regression equation.

Sample ID	Biological Sex	Real Age	Histological estimated age
ABHT-1	Female	29	28.50
ABHT-2	Female	39	40.06
ABHT-3	Female	45	40.46
ABHT-4	Female	49	43.18
ABHT-6	Male	39	40.84
ABHT-7	Male	35	38.41
C Athi 03	Male	43	46.01
C Athi 05	Male	44	45.80
C_Athi_06	Male	7	6.66
C Athi 08	Female	71	75.10
C Athi 09	Female	86	82.07
C Athi 10	Male	45	44.77

**Table 5:** Real ages of the test series modern individuals in comparison with the estimated ages from the HD regression equation.

Sample ID	Biological Sex	Real Age	Histological estimated age
CAthi 11	Male	51	45.50
C Athi 13	Male	79	44.97
C Athi 14	Female	82	63.96
C Athi 15	Male	58	38.09
C Athi 16	Male	49	44.73
C_Athi_17	Male	47	44.02

**Table 6:** Estimated age ranges of the of modern additional individuals in comparison with the estimated ages from the HD regression equation.

Sample ID	Biological Sex	Age range	Histological estimated age
ABHT-5	Female	45–49	45.17
ABHT-8	Female	50–59	45.98
C Alhi 03	Male	25–45	43.83
C_Alhi_04(1)*	Male	30–45	70.60
C Alhi 04(2)	Female	35–50	48.21
C_Alhi_05	Male	35–45	38.85
C_Alhi_06	Male	20–40	42.31

* Individual suffering from ankylosis spondylitis.

**Table 7:** Estimated ages of the individuals of ancient Thessaloniki by the application of the standard anthropological methods and the HD regression equation.

Sample ID	Biological Sex	Age range	Histological estimated age
METh 03	Male	20–35	40.87
METh 04	Male	34–44	44.00
METh 05	Female	35–40	39.38
METh 08	Female	20–25	41.78
METh 10	Male	35–50	43.49
METh 13	Female	30–35	38.03
METh_15	Male	20–35	34.36
METh 17	Male	30–35	40.82
METh 33	Female	35–50	39.44
METh 34	Male	35–50	38.02
METh 35	Male	35–40	46.06
METh 36	Male	35–40	37.91
METh 37	Male	35–50	42.36
METh 38	Female	35–50	42.03
METh 39	Female	35–50	43.90
METh 40	Female	35–50	46.08
METh 53	Male	25–35	32.52
METh 54	Female	21–25	45.06
METh 55	Male	25–50	41.95
METh 56.3	Female	30–45	44.22
METh 58.1	Female	50–60	38.06
METh 58.2	Female	35–50	42.93
METh 58.3	Female	35–40	45.73
METh 74	Female	25–35	45.77
METh 75	Male	40–50	43.66

## Data Availability

All necessary data are included in the Supplementary Information at the Attached Files

## References

[R1] Acheilara L, Adam-Veleni P, Tzanavari K (2007). The Archaeological Work in Macedonia and Thrace.

[R2] Adam-Veleni P, Adam-Veleni P, Terzopoulou D (2012). FieldHouseGardenGrave.

[R3] Agnew AM, Betsinger TK, Justus HM (2015). Post-cranial traumatic injury patterns in two medieval polish populations: The effects of lifestyle differences. PLoS One.

[R4] Andronowski JM, Crowder C, Soto-Martinez M (2018). Recent advancements in the analysis of bone microstructure: New dimensions in forensic anthropology. Forensic Sci Res.

[R5] Barger-Lux MJ, Recker RR (2002). Bone Microstructure in Osteoporosis: Transilial Biopsy and Histomorphometry. Top Magn Reson Imaging.

[R6] Bell KL, Loveridge N, Reeve J, Thomas CDL, Feik SA, Clement JG (2001). Super-osteons (remodeling clusters) in the cortex of the femoral shaft: Influence of age and gender. Anat Rec.

[R7] Bell LS (1990). Palaeopathology and diagenesis: An SEM evaluation of structural changes using backscattered electron imaging. J Archaeol Sci.

[R8] Bell LS, Skinner MF, Jones SJ (1996). The speed of post mortem change to the human skeleton and its taphonomic significance. Forensic Sci Int.

[R9] Bonicelli A, Zioupos P, Arnold E, Rogers KD, Xhemali B, Kranioti EF (2021). Age related changes of rib cortical bone matrix and the application to forensic age-at-death estimation. Sci Rep.

[R10] Booth T, Errickson D, Thompson T (2017). Human Remains: Another Dimension.

[R11] Botha D, Steyn M, Lynnerup N (2019). Histological age-at-death estimation in white South Africans using stereology. Int J Leg Med.

[R12] Bouvier M, Ubelaker DH (1977). A comparison of two methods for the microscopic determination of age at death. Am J Phys Anthropol.

[R13] Brooks S, Suchey JM (1990). Skeletal age determination based on the os pubis: A comparison of the Acsádi-Nemeskéri and Suchey-Brooks methods. Hum Evol.

[R14] Brothwell DR, Brothwell DR (1981). Digging up Bones: the Excavation, Treatment, and Study of Human Skeletal Remains.

[R15] Buikstra J, Ubelaker D (1994). Standards for Data Collection from Human Skeletal Remains. Arkansas Archeol Surv Res Series.

[R16] Burr DB, Ruff CB, Thompson DD (1990). Patterns of skeletal histologic change through time: Comparison of an archaic native american population with modern populations. Anat Rec.

[R17] Canty A, Ripley BD (2022). boot: Bootstrap R (S-Plus) Functions.

[R18] Caruso V, Cummaudo M, Maderna E, Cappella A, Caudullo G, Scarpulla V, Cattaneo C (2018). A comparative analysis of microscopic alterations in modern and ancient undecalcified and decalcified dry bones. Am J Phys Anthropol.

[R19] Chan AHW, Crowder CM, Rogers TL (2007). Variation in cortical bone histology within the human femur and its impact on estimating age at death. Am J Phys Anthropol.

[R20] Chen H, Zhou X, Fujita H, Onozuka M, Kubo K-Y (2013). Age-related changes in trabecular and cortical bone microstructure. Int J Endocrinol.

[R21] Cho H, Stout SD, Madsen RW, Streeter MA (2002). Population-specific histological age-estimating method: a model for known African-American and European-American Skeletal Remains. J Forensic Sci.

[R22] Consensus development conference (1993). Diagnosis, prophylaxis, and treatment of osteoporosis. Am J Med.

[R23] Cooke KM, Mahoney P, Miszkiewicz JJ (2022). Secondary osteon variants and remodeling in human bone. Anat Rec.

[R24] Crowder C, Dominguez VM, Heinrich J, Pinto D, Mavroudas S (2022). Analysis of histomorphometric variables: Proposal and validation of osteon definitions. J Forensic Sci.

[R25] Crowder C, Heinrich J, Stout SD (2012). Rib histomorphometry for adult age estimation. Methods Mol Biol (Clifton N.J.).

[R26] Cunningham CA, Black SM, Payne-James J, Byard RW (2016). Encyclopedia of Forensic and Legal Medicine.

[R27] Currey JD (1964). Some effects of ageing in human Haversian systems. J Anat.

[R28] Curtis JM, Nawrocki SP, Latham KE, Finnegan M (2010). Age Estimation of the Human Skeleton.

[R29] Dominguez VM, Mavroudas S, Adserias-Garriga J (2019a). Age Estimation: A Multidisciplinary Approach.

[R30] Dominguez VM, Mavroudas S, Adserias-Garriga J (2019). Age Estimation.

[R31] Dominquez VM, Agnew AM (2016). Examination of Factors Potentially Influencing Osteon Size in the Human Rib. Anatomical Rec.

[R32] Drusini A (1987). Refinements of two methods for the histomorphometric determination of age in human bone. Z Morphol Anthropol.

[R33] Dudar J, Pfeiffer S, Saunders S (1993). Evaluation of morphological and histological adult skeletal age-at-death estimation techniques using ribs. J Forensic Sci.

[R34] Ericksen MF (1991). Histologic estimation of age at death using the anterior cortex of the femur. Am J Phys Anthropol.

[R35] Ferembach D, Schwindezky I, Stoukal M (1980). Recommendation for Age and Sex Diagnoses of Skeletons. J Hum Evol.

[R36] Franklin D (2010). Forensic age estimation in human skeletal remains: Current concepts and future directions. Leg Med.

[R37] Frost HM (1987). Secondary osteon populations: An algorithm for determining mean bone tissue age. Am J Phys Anthropol.

[R38] García-Donas JG, Dyke J, Paine RR, Nathena D, Kranioti EF (2016). Accuracy and sampling error of two age estimation techniques using rib histomorphometry on a modern sample. J Forensic Leg Med.

[R39] García-Donas JG, Paine RR, Bonicelli A, Kranioti EF (2022). Age estimation for two Mediterranean populations: Rib histomorphometry applied to forensic identification and bone remodelling research. Int J Leg Med.

[R40] Goliath JR, Stewart MC, Stout SD (2016). Variation in osteon histomorphometrics and their impact on age-at-death estimation in older individuals. Forensic Sci Int.

[R41] Hackett CJ (1981). Microscopical Focal Destruction (Tunnels) in Exhumed Human Bones. Med Sci Law.

[R42] Işcan MY, Loth SR, Wright RK (1985). Age estimation from the rib by phase analysis: White females. J Forensic Sci.

[R43] Iwaniec UT, Crenshaw TD, Schoeninger MJ, Stout SD, Ericksen MF (1998). Methods for improving the efficiency of estimating total osteon density in the human anterior mid-diaphyseal femur. Am J Phys Anthropol.

[R44] Jans MME, Pokines JT, Symes SA (2014). Manual of Forensic Taphonomy.

[R45] Jans MME, Nielsen-Marsh CM, Smith CI, Collins MJ, Kars H (2004). Characterisation of microbial attack on archaeological bone. J Archaeol Sci.

[R46] Jenkins LL, Burg KJL, An YH, Martin KL (2003). Handbook of Histology Methods for Bone and Cartilage.

[R47] Ju Y-I, Sone T (2021). Effects of different types of mechanical loading on trabecular bone microarchitecture in rats. J Bone Metabol.

[R48] Kakasa C, Aidonis A, Bantavanou P, Souleles A, Ganiatsou E, Papageorgopoulou C, Chrysostomou A, Chrysostomou P, Saripanidi V The West Cemetery at Archontiko near Pella 1 The First Chronological Phase (Early Iron Age—Early 6th c. BC).

[R49] Karydi C, García-Donas JG, Tsiminikaki K, Bonicelli A, Moraitis K, Kranioti EF (2022). Estimation of Age-at-Death Using Cortical Bone Histomorphometry of the Rib and Femur: A Validation Study on a British Population. Biology.

[R50] Keough N (2007). Estimation of age at death from the microscopic structure of the femur.

[R51] Kerley ER (1965). The microscopic determination of age in human bone. Am J Phys Anthropol.

[R52] Khan I, Jamil MMA, Nor FM (2017). Evaluation and reliability of bone histological age estimation methods. J Fundamental Appl Sci.

[R53] Khurana JS, Fitzpatrick LA, Khurana JS (2009). Bone Pathology.

[R54] Kontopoulos I, Penkman K, Liritzis I, Collins MJ (2019). Bone diagenesis in a Mycenaean secondary burial (Kastrouli, Greece. Archaeol Anthropol Sci.

[R55] Lovejoy CO, Meindl RS, Mensforth RP, Barton TJ (1985a). Multifactorial determination of skeletal age at death: A method and blind tests of its accuracy. Am J Phys Anthropol.

[R56] Lovejoy CO, Meindl RS, Pryzbeck TR, Mensforth RP (1985b). Chronological metamorphosis of the auricular surface of the ilium: A new method for the determination of adult skeletal age at death. Am J Phys Anthropol.

[R57] Maat GJR, Maes A, Aarents MJ, Nagelkerke NJD (2006). Histological age prediction from the femur in a contemporary Dutch sample. J Forensic Sci.

[R58] Maat GJR, Van Den Bos RPM, Aarents M (2001). Manual preparation of ground sections for the microscopy of natural bone tissue: Update and modification of Frost’s ‘rapid manual method. Int J Osteoarchaeol.

[R59] Maggio A, Franklin D (2019). Histomorphometric age estimation from the femoral cortex: A test of three methods in an Australian population. Forensic Sci Int.

[R60] Maggio A, Franklin D (2021). An examination of histomorphometric relationships in the anterior and posterior human femoral cortex. J Bone Miner Metab.

[R61] Martin RB, Pickett JC, Zinaich S (1980). Studies of skeletal remodeling in aging men. Clin Orthop Relat Res.

[R62] Matsuo H, Tsurumoto T, Maeda J, Saiki K, Okamoto K, Ogami-Takamura K, Kondo H, Tomita M, Yonekura A, Osaki M (2019). Investigating interindividual variations in cortical bone quality: Analysis of the morphotypes of secondary osteons and their population densities in the human femoral diaphysis. Anat Sci Int.

[R63] Mavroudas SR, Alfsdotter C, Bricking A, Madgwick R (2023). Experimental investigation of histotaphonomic changes in human bone from whole-body donors demonstrates limited effects of early post-mortem change in bone. J Archaeol Sci.

[R64] Miles AE (1962). Assessment of the Ages of a Population of Anglo-Saxons from Their Dentitions. Proc R Soc Med.

[R65] Miszkiewicz JJ (2016). Investigating histomorphometric relationships at the human femoral midshaft in a biomechanical context. J Bone Miner Metab.

[R66] Miszkiewicz JJ, Mahoney P (2016). Ancient Human Bone Microstructure in Medieval England: Comparisons between Two Socio-Economic Groups. Anat Rec.

[R67] Mulhern DM, Van Gerven DP (1997). Patterns of femoral bone remodeling dynamics in a medieval Nubian population. Am J Phys Anthropol.

[R68] Nigil Haroon N, Szabo E, Raboud JM, Mcdonald-Blumer H, Fung L, Josse RG, Inman RD, Cheung AM (2015). Alterations of bone mineral density, bone microarchitecture and strength in patients with ankylosing spondylitis: A cross-sectional study using high-resolution peripheral quantitative computerized tomography and finite element analysis. Arthritis Res Ther.

[R69] Nikita E (2020). Documented skeletal collections in Greece: Composition, research, and future prospects. Am J Phys Anthropol.

[R70] Ortner DJ (1975). Aging effects on osteon remodeling. Calcif Tissue Res.

[R71] Ott SM, Bilezikian JP, Raisz LG, Rodan GA (2002). Principles of Bone Biology.

[R72] Parfitt AM (1984). Age-related structural changes in trabecular and cortical bone: Cellular mechanisms and biomechanical consequences. Calcif Tissue Int.

[R73] Pavón MV, Cucina A, Tiesler V (2010). New formulas to estimate age at death in maya populations using histomorphological changes in the fourth human rib*. J Forensic Sci.

[R74] Pfeiffer S (1992). Cortical bone age estimates from historically known adults. Zeitschrift Für Morphologie Und Anthropologie.

[R75] Pfeiffer S, Crowder C, Harrington L, Brown M (2006). Secondary Osteon and Haversian canal dimensions as behavioral indicators. Am J Phys Anthropol.

[R76] Phenice TW (1969). A newly developed visual method of sexing the os pubis. Am J Phys Anthropol.

[R77] Pitfield R, Miszkiewicz JJ, Mahoney P (2017). Cortical Histomorphometry of the Human Humerus During Ontogeny. Calcif Tissue Int.

[R78] R Core Team (2023). R: A Language and Environment for Statistical Computing.

[R79] Richman EA, Ortner DJ, Schulter-Ellis FP (1979). Differences in intracortical bone remodeling in three aboriginal American populations: Possible dietary factors. Calcif Tissue Int.

[R80] Ries WL, An YH, Martin KL (2003). Handbook of Histology Methods for Bone and Cartilage.

[R81] Robling AG, Castillo AB, Turner CH (2006). Biomechanical ans molecular regulation of bone remodeling. Annu Rev Biomed Eng.

[R82] Robling AG, Stout SD (2008). Biological Anthropology of the Human Skeleton.

[R83] Ruff CB, Hayes WC (1988). Sex differences in age-related remodeling of the femur and tibia. J Orthop Res.

[R84] Schindelin J, Arganda-Carreras I, Frise E, Kaynig V, Longair M, Pietzsch T, Preibisch S, Rueden C, Saalfeld S, Schmid B, Tinevez J-Y (2012). Fiji: An open-source platform for biological-image analysis. Nat Methods.

[R85] Seeman E (2008). Bone quality: The material and structural basis of bone strength. J Bone Miner Metab.

[R86] Singh IJ, Gunberg DL (1970). Estimation of age at death in human males from quantitative histology of bone fragments. Am J Phys Anthropol.

[R87] Sobol J, Ptaszyńska-Sarosiek I, Charuta A, Oklota-Horba M, Żaba C, Niemcunowicz-Janica A (2015). Estimation of age at death: Examination of variation in cortical bone histology within the human clavicle. Folia Morphol.

[R88] Soueref K (2011). Topographicals and Archaeologicals of Central Macedonia.

[R89] Stout SD, Crowder C, Stout SD, Crowder C (2012). Bone Histology: an Anthropological Perspective.

[R90] Stout SD, Dietze WH, Işcan M, Loth SR (1994). Estimation of Age at Death Using Cortical Histomorphometry of the Sternal End of the Fourth Rib. J Forensic Sci.

[R91] Stout SD, Gehlert SJ (1980). The relative accuracy and reliability of histological aging methods. Forensic Sci Int.

[R92] Stout SD, Lueck R (1995). Bone remodeling rates and skeletal maturation in three archaeological skeletal populations. Am J Phys Anthropol.

[R93] Stout S, Paine RR (1992). Brief communication: Histological age estimation using rib and clavicle. Am J Phys Anthropol.

[R94] Suzuki S, Maggiano IS (2018). Histological age assessment in a prehispanic Maya sample from Xcambó, Yucatan, Mexico: Benefits and limitations. J Archaeol Sci Rep.

[R95] Thompson DD (1980). Age changes in bone mineralization, cortical thickness, and Haversian canal area. Calcif Tissue Int.

[R96] Todd TW (1920). Age changes in the pubic bone. I. The male white pubis. Am J Phys Anthropol.

[R97] Tompson DD (1979). The core technique in the determination of age at death of skeletons. J Forensic Sci.

[R98] Tsimpidou-Avloniti M, Adam-Veleni P, Tzanavari K (2007). The Archaeological Work in Macedonia and Thrace.

[R99] Turner-Walker G (2019). Light at the end of the tunnels? The origins of microbial bioerosion in mineralised collagen. Palaeogeogr Palaeoclimatol Palaeoecol.

[R100] Uytterschaut HT (1985). Determination of skeletal age by histological methods. Z Morphol Anthropol.

[R101] Valakopoulos EA (1983). History of Thessaloniki 316 B.C - 1983.

[R102] Wang F, Metzner F, Osterhoff G, Schleifenbaum S (2022). Assessment of the efficiency of different chemical treatments and ultrasonic cleaning for defatting of cancellous bone samples. Cell Tissue Bank.

[R103] Yeni YN, Wu B, Huang L, Oravec D (2013). Mechanical loading causes detectable changes in morphometric measures of trabecular structure in human cancellous bone. J Biomech Eng.

[R104] Yerramshetty J, Akkus O, Silva MJ (2013). Skeletal Aging and Osteoporosis: Biomechanics and Mechanobiology.

[R105] Zhang X, Aubin JE, Inman RD (2003). Molecular and cellular biology of new bone formation: Insights into the ankylosis of ankylosing spondylitis. Curr Opin Rheumatol.

