# Supplementary material for: Histomorphometric method for age-at-death estimation using the anterior side of the femoral midshaft
Source: J Archaeol Sci Rep. Author manuscript; Available in PMC 2026 Aug 9. (PMC7619337; doi:10.1016/j.jasrep.2025.105012)
Supplement: Supplementary file 1 [file EMS217084-supplement-_Supplementary_Material.zip › 1-s2.0-S2352409X25000446-mmc1.docx]

**Table S1**. Summarized information of histomorphological methods for estimating age on modern and inhumed skeletal material including the characteristics of the datasets, the variables used and the field of view as well as the resulted standard estimation error of the regression equations. In the figures the area of sampling on the bones and the microscopical fields of the sections studied are presented with red lines and dotes respectively.

| **Reference** | **Bone sampling and histological method** | **Dataset characteristics** | **Variables used** | **Reported accuracy** |
| --- | --- | --- | --- | --- |
| Kerley (1965) |  | 88 male και 29 female individuals of common ancestry. Age range 0 (stillborn) to 95 years old.    Additional 56 individuals used as testers for the regression equations.  Archaeological material, coming from Philippines, Aleutian Islands, Kentucky, Virginia and Florida, used to test the applicability of the method on other materials. | 2,06mm^2^ area field of view.  1,62mm diameter field of view  **1**. **Total number of intact osteons**  **2. Total number of osteon fragments**  **3. Percentage of circumferential lamellar bone**  **4. Total number of non-Haversian canal** | Standard deviation: ±10.54  Standard estimation error: ±5,27 years |
| Ahlqvist & Damsten (1969) |  | 20 cross-sections | Optical grid of100 squares  1. **Percentage of intact osteons**  2. **Percentage of osteon fragments**  3. **Percentage of non-Haversian canal**  4. **Percentage of circumferential lamellar bone** | Standard estimation error: ± 6.71years |
| Singh & Gunberg (1970) |  | 59 male cadavers of Anatomy dissecting room. 40 samples removed from all the three bones, while the rest 19 only from mandible.  7 females were, additionally, added as testers for the regression equation. | Block of 1cm x 1cm  2,0mm^2^ area field of view.  Two random fields of view.  1. **Total number of osteons**  2. **Average number of lamellae**  3. **Average short diameter of Haversian canal** | Standard estimation error: ±5,16 years |
| Bouvier & Ubelaker (1977) |  | 67 samples, a part of it (40 samples) comes from the study of Kerley (1965). | 1,45mm diameter field of view  1. **Percentage of remodelled bone (Ahlqvist-Damsten’s)**  2. **Total number of intact osteons (Kerley’s)** | Standard estimation error **Kerley (1965)** ±8.20 years, **Alqvist & Damsten (1969)** ±9.50 years |
| Thompson (1980) |  | 64 male and 52 female cadavers, with age range over 28 years.  Additional 8 individuals from forensic cases as testers for the regression equation. | 0.992 mm^2^ area of study  Sampling of cortical bone cores of 0,4cm  19 variables from which the most accurate:  1. **Haversian canal total area (mm^2^ / field)**  2. **Total number of Haversian canal (#/filed)**  3. **Average Haversian canal area (mm^2^/field)** | Standard estimation error: ±6,21 years |
| Drusini (1987) |  | 15 males and 9 females, with age range 19-50 years, coming from the Osteological collection of the Anthropological Institute of University of Padua | 1,68mm diameter field of view (correction factor 0,93).  0.9025 mm^2^ area of study  Kerley:  1. **Total number of intact osteons**  2. **Total number of osteon fragments**  Thompson:   1. **Density of intact osteons, Haversian and non-Haversian canals** | Standard estimation error: ±3.92 years |
| Ericksen (1991) |  | 159 male and 125 female cadavers coming from the Anatomy dissecting room of the Medical University George Washington  Additional material of 31 males and 27 females from two modern cemeteries from Dominican Republic, and 6 individuals coming from autopsies. | Merz: standard counting reticule for area measurement and portrait of the microscopic field.  1. **Cortical area**  2. **Intact osteon density**  3. **Fragmentary osteon density**  4. **Total visible osteon density** | Standard estimation error: rib ±3,9 years, clavicle ±5,91 years, combination of bones ±3,8 years |
| Stout & Paine (1992) |  | 7 females and 32 males, with age range 13-62 years (32 white, 4 coloured and 3 of unknown ethnicity), coming from autopsies.  Additional samples of 12 ribs and 7 clavicles of white individuals from autopsies and forensic cases. | Merz: standard counting reticule for area measurement and portrait of the microscopic field.  1. **Cortical area**  2. **Intact osteon density**  3. **Fragmentary osteon density**  4. **Total visible osteon density** | Standard estimation error: rib ±3,9 years, clavicle ±5,91 years, combination of bones ±3,8 years |
| Dudar et al. (1993) |  | 31 males and 19 females, with age range 17,5-95 years originating from anatomy teaching facilities of south Ontario.  Additional archaeological material from cemeteries on Harvie site, and the historic St. Thomas Anglican church of Belleville. | Merz standard counting reticule    1. **Quantification of cortical cross-sectional area**  2. **Total remodeled bone unit counts**  3. **Calculation of visible osteon creations** | Standard deviation: ±17,4 years  Standard estimation error: ±11, years |
| Stout et al. (1994) |  | 60 individuals, with age range 11-88 years, from autopsies. | 0,37mm^2^ area field of view.  1. **Intact osteon density**  2. **Fragmentary osteon density**  3. **Osteon population density** | Standard estimation error: rib ±10.43 years, combination of two bones ±7.18 years |
| Cho et al. (2002) |  | 103 African-American individuals, with age range 17-95 years, and 51 European-American individuals, with age range 17-82 years, from autopsies and forensic cases. Part of the sample comes from the study of Stout & Paine (1994). | Method of Stout & Paine (1994)    1. **Mean osteonal cross-sectional area per mm^2^**  2. **Intact osteon density per mm^2^**  3. **Fragmentary osteon density per mm^2^**  4. **Osteon population density per mm^2^**  5. **Relative cortical area** | Standard estimation error: ±15.14 years |
| Pavón et al. (2010) |  | 34 males and 2 females, with age range 20-87 years coming from autopsies of modern population of Maya origin.  Additional 13 individuals as testers for the regression equation | Merz counting reticule of 6 X6   1. **Osteon population density** 2. **Calculation of cortical area** 3. **Average osteon size** | Standard estimation error: ±6,5 years |
| Curtis & Nawrocki (2010) |  | 57 modern cadavers, with age range 29-99 years, coming from necropsies of the University of Indiana, University of Indianapolis, Medical and Dental faculty of Indiana and University of Indiana at Bloomington.  Additional 8 individuals from the University of Cincinnati and 27 individuals from the Scientific Helth Center in Syracuse of New York. | Bone cores from the centre of left or right frontal eminence.    1. **Percentage of intact osteons per mm^2^**  2. **Percentage of osteonal area per mm^2^**  3. **Osteon population density**  4. **Thickness of the external table (μm)** | Standard estimation error: males ±11,16 years, females ±10,31 years |

**Figure S1**. Scatter plot showing the distribution of secondary osteon density values according to the real ages of all modern individuals. Pink circles indicate female individuals, while grey dots indicate the male individuals. This figure shows the intense scattering of male individuals over 60 years old affecting the function of the regression model (blue line).

**Figure S2**. Scatter plot showing the distribution of osteon fragment density values according to the real ages of all modern individuals. Pink circles indicate female individuals, while grey dots indicate the male individuals. This figure shows the intense scattering of male individuals over 60 years old affecting the function of the regression model (blue line).

**Figure S3**. Scatter plot showing the distribution of osteon density values according to the real ages of all modern individuals. Pink circles indicate female individuals, while grey dot indicates the male individuals. This figure shows the intense scattering of male individuals over 60 years old affecting the function of the regression model (blue line).

**Figure S4**. Scatter plot showing the distribution of the average number of secondary osteons per 1mm^2^ according to the real ages of all modern individuals. Pink circles indicate female individuals, while the grey dots indicate the male individuals.

**Figure** **S5**. Scatter plot showing the distribution of the average number of osteon fragments per 1mm^2^ according to the real ages of all modern individuals. Pink circles indicate female individuals, while the grey dots indicate the male individuals.

**Figure S6**. Micro-images captured with digital microscope camera Axiocam ICc3 from Axioscope A.1 microscope with magnification X100. **A**. Microstructure of a subadult male individual (C_Athi_06) (7 years old) with large and irregular osteons. **B**. Microstructure of a male individual (ABHT-6) (39 years old) with medium and small size osteons more round-like and larger haversian canals. **C**. Microstructure of a female individual (C_Athi_08) (71 years old) with small size and quite circulated osteons.

S1. Example of histomorphometric method application of OPD regression equation

**Figure S7**. Micro-image of a male individual (C_Athi_10) 45 years old from the Forensic Unit of Athens, captured with digital microscope camera Axiocam ICc3 from Axioscope A.1 microscope with magnification X100. The images 1, 2, 3 show the steps of the histomorphometric method described below.

Micro-images (like Figure 1.1) from 10 random locations of the endosteum were captured and studied. The micro-images were imported in Fiji software and a grid of 1mm^2^ area with centred squares was applied (Figure 1.2). Each square occupied an area of 0.0121μm^2^ or (0.11μm)^2^.

At each micro-image we counted: 1) the secondary osteons (SO), 2) the osteon fragments (OF) and 3) the intersections that included and osculate each microstructure respectively. In Figure 1.2 the yellow dots mark the intersections of the secondary osteons, while in Figure 1.3 the orange dots mark the intersections of the osteon fragments. The coloured squares and triangles show the area that the secondary osteons and osteon fragments occupy, respectively. After completing the counting in all 10 micro-images we sum the number of secondary osteons and the osteon fragments independently and combined (Table S3). To calculate the area of occupation of the microstructures we summed the number of intersections and multiplied the result with the square area 0.0121μm^2^ (Formula I). Then the area was divided by the total number of each microstructure or the combination of these resulting the density per/mm^2^ (Formula 2). The calculated densities were then applied on the regression equations. According to the results of the OPD non-linear cubic regression equation (Formula 3) the individual was estimated at 47.77 years old, that is two years apart from his real age.

**Table S3**. The table includes all the necessary numbers for the are-at-death estimation resulting from the histomorphometric method. Total and per location number of secondary osteons (SO), osteon fragments (FO), combination of osteons (SO + FO), Intersections and the resulted Area and Density.

| **Microscopic location** | **SO** | **FO** | **SO Intersections** | **FO intersections** | **SO + FO intersections** |
| --- | --- | --- | --- | --- | --- |
| 0 | 6 | 7 | 47 | 46 | 93 |
| 1 | 8 | 9 | 53 | 51 | 104 |
| 2 | 10 | 9 | 55 | 52 | 107 |
| 3 | 4 | 9 | 35 | 57 | 92 |
| 4 | 7 | 7 | 46 | 53 | 99 |
| 7 | 6 | 7 | 38 | 47 | 85 |
| 8 | 7 | 6 | 49 | 47 | 96 |
| 11 | 9 | 7 | 57 | 45 | 102 |
| 23 | 5 | 6 | 37 | 50 | 87 |
| 26 | 9 | 7 | 44 | 35 | 79 |
| **Total** | **71** | **74** | 461 | 483 | 944 |
| Occupied area (μm^2^/1mm^2^) | | | 5.5781 | 5.8443 | 11.4224 |
| **Density #/mm^2^** | | | **12.72835** | **12.66191** | **12.69435** |

| Formula 1: | SO area = 461 (total intersections) * 0.0121 = 5.5781 |
| --- | --- |
|  | FO area = 483 (total intersections) * 0.0121 = 5.8443 |
|  | Total area = 944 (total intersections) * 0.0121 = 11.4224 |

| Formula 2: | SPD = 71 (total number SO) / 5.5781 (SO area) = 12.72835 |
| --- | --- |
|  | FPD = 74 (total number FO) / 5.8443 (FO area) = 12.66191 |
|  | OPD = 145 (total number FO + SO) / 11.4224 (total osteon area) = 12.69435 |

| Formula 3: | Age = 290.65337*12.69435 - (19.48360 * 12.69435^2^) + (0.42995 * 12.69435^3^) - 1384.67423 = **47.77** | Real age = **45** |
| --- | --- | --- |
